# Localized Scleroderma Cutaneous Assessment Tool (LoSCAT) adapted for use in adult patients: report from an initial validation study

**DOI:** 10.1186/s12955-018-1010-z

**Published:** 2018-09-14

**Authors:** Alina Skrzypek-Salamon, Anna Lis-Święty, Irmina Ranosz-Janicka, Ligia Brzezińska-Wcisło

**Affiliations:** Department of Dermatology, School of Medicine in Katowice, Medical University of Silesia, Francuska Str. 20/24, 40-027 Katowice, Poland

**Keywords:** Adult-onset morphea, Outcome measure, Skin score, Quality of life

## Abstract

**Background:**

Localized scleroderma (LoS) affects both children and adults and is associated with permanent functional and cosmetic impairment, and reduced quality of life predominating in adults. The Localized Scleroderma Cutaneous Assessment Tool (LoSCAT) is a clinical instrument designed to measure an activity and damage of LoS. It has been validated for use with pediatric LoS patients. This study assessed the validity and reliability of the LoSCAT adapted for use in adults.

**Methods:**

Before the initiation of the study two examiners participated in an intensive training course carried out by an expert in LoS. Appendices describing each LoSCAT domain were prepared. Features determining disease activity and damage in adult LoS patients were identified to properly evaluate the physician (Phys) and patient (Pt) global assessment (GA) of disease activity (A)/severity (S) and damage (D), which were used to assess convergent validity of the LoSCAT. Correlations of physician- and patient-derivied measures with Skindex-29 were also analysed.

**Results:**

The study included 40 adult LoS patients (33 females and 7 males) with different subtypes of LoS. Intra and inter-rater reliability of the LoSCAT was found to be excellent. Positive correlations were observed between the PhysGA-A, PhysGA-D, PtGA-A and the LoSCAT’s domains, while no correlations between them and the PtGA-D were found. There were no relationships between LoSCAT’s components and Skindex-29.

**Conclusions:**

Despite the LoSCAT is a reliable tool for an assessment of cutaneous lesions, additional health status instruments are necessary to a holistic approach to LoS in adults.

## Background

Localized scleroderma (LoS) is a chronic connective tissue disorder characterised by sclerosis of the skin and, in some cases, deeper tissues (subcutaneous fat, muscles or bones). The disease affects both children and adults, with the linear subtype predominating in children and the plaque and generalised subtypes predominating in adults [1]. LoS lesions, especially in the linear and generalised forms, are associated with permanent functional and cosmetic impairment [1]. Recurrences of the disease may occur and are independent of its onset mode [1]. Active lesions require anti-inflammatory or immunosuppressive management to limit further progression of the disease. Diagnosis is made on the basis of clinical features and, when necessary, biopsy specimen. There are no adequate laboratory tests available to precisely assess disease activity or progression. Thermography, ultrasound, Doppler flowmetry or magnetic resonance imaging are useful tools for LoS assessing, but they are valuable only in the hands of an experienced physician and are not easy accessible in daily clinical practice [2]. A few scoring systems have been proposed for this purpose: the visual analogue scale (VAS), the computerised skin score, the DIET score (D, dyspigmentation/hyperpigmentation; I, induration; E, erythema and T, telangiectasias) and the Localized Scleroderma Cutaneous Assessment Tool (LoSCAT) [3–8]. The LoSCAT is the first clinician-report measure that differentiates between disease activity and damage, because it consists of two components: the Modified Localized Scleroderma Skin Severity Index (mLoSSI), which is designed to measure LoS activity, and the Localized Scleroderma Skin Damage Index (LoSDI), which measures the damage associated with LoS [3, 4]. Both indexes are based on physical examination of LoS skin lesions in 18 cutaneous anatomic sites. The LoSCAT was initially validated in juvenile LoS. The sensitivity of the scale (especially the mLoSSI) to the evolution of the disease as a result of the therapy used was confirmed by some authors [2, 9, 10].

Because there are known clinical features that differentiate juvenile from adult-onset LoS [1], the LoSCAT, in our opinion, requires proper validation testing in adults. The purpose of this paper was to initially validate the LoSCAT adapted for use in adult patients with LoS.

## Methods

### Patients

Study participants were recruited from the Dermatology Department at the Medical University of Silesia in Katowice, Poland, in the period between 1st January and 31st December of the 2017. All study protocols were formally approved by the ethics committee of the Medical University of Silesia (ref. KNW/0022/KB1/134/15). Prior to their inclusion in the study, all patients provided informed consent. Patients with LoS were chosen by the attending clinician to represent a range of disease severity (mild to severe), gender (female and male) and age (over 18 years of age). The diagnosis was based on the clinical criteria and, when necessary, confirmed with a histology test. LoS was classified according to the German guidelines proposed by Kreuter et al. [11] Exclusion criteria were as follows: 1) patients under 18 years old, 2) pregnancy or breastfeeding, 3) the presence of a scleroderma-like disorders, 4) incomplete patient information. All the patients were taken through a detailed medical history and physical examination. The general data of enrolled patients, including demographic, epidemiologic and clinical data, were recorded (specified in Table 1).

**Table 1:** Demographic and clinical characteristics of localized scleroderma patients

Variable	No	SD	Range
Females: Males	33:7		
Variables	Mean	SD	Range
Age (in years)	49.05	18.34	19–81
Age at disease onset (in years)	42.43	19.37	4–75
Duration of disease (in years)	6.63	8.31	0,3–30
mLoSSI	7.15	7.21	0–29
LoSDI	10.43	9.82	0–52
PhysGA-A	31.35	21.58	0–76
PhysGA-D	39.2	15.42	0–88
PtGA-S	42.48	31.42	0–100
PtGA-D	47.63	26.83	0–100
Variables	No. of patients	Percent	
Active disease	35	87.5	
Without remission	26	65	
Reccurent disease 1st relapse	6	15	
Reccurent disease 2nd relapse	3	7.5	
Inactive disease	5	12.5	
Subtypes			
Plaque	18	45	
Atrophoderma of Pasini and Pierini	8	20	
Generalized	7	17.5	
Mixed	4	10	
Linear	3	7.5	
Musculoskeletal manifestations	3	7.5	
Comorbid medical conditions	25	62.5	
Hypertensio arterialis	9	22.5	
Diabetes type 2	5	12.5	
Gastroesophageal reflux	3	7.5	
Hashimoto disease	2	5	
Hand eczema	2	5	
Rheumatoid arthritis	2	5	
Other	5	12.5	

*SD* standard deviation, *mLoSSI* modified Localized Scleroderma Skin Severity Index, *LoSDI* Localized Scleroderma Damage Index, *PhysGA-A* physician global assessment of disease activity, *PhysGA-D* physician global assessment of disease damage, *PtGA-S* patient global assessment of disease severity, *PtGA-D* patient global assessment of disease damage

### Performance characteristics of health status instruments

Because of the survey design, the following tools for determining activity and damage in LoS were used: the mLoSSI, the LoSDI, the patient and physician global assessment of disease (PtGA, PhysGA) on the VAS [3, 4, 8]. An additional investigation using Skindex-29 was conducted to analyze potential relationship of physician- and patient-derivied scores to quality of life (QoL) measures [12, 13].

The mLoSSI and LoSDI assess LoS skin lesions in 18 anatomic sites: head, neck, chest, abdomen, upper back, lower back and the right and left arms, forearms, hands/fingers, buttocks/thighs, legs and feet. Both indexes are a total sum of the score given in several domains. In each of the cutaneous anatomic sites, the mLoSSI assesses the following domains: new lesion/lesion extension, erythema and skin thickness; the LoSDI assesses dermal atrophy, subcutaneous atrophy and dyspigmentation in each of the sites. The arising of a new lesion or the enlargement of an existing lesion within the previous month is scored 3. Other domains of LoSCAT (erythema, skin thickness, dermal atrophy, subcutaneous atrophy and dyspigmentation) are scored using a unique 4-point scale ranging from 0 (‘no disability’) to 3 (‘completely disabled’). Higher mLoSSI scores indicate higher activity of the disease, whereas higher LoSDI scores represent greater damage caused by the disease [3, 4, 8].

The GAs of the disease, typically used in association with the LoSCAT, are rating scales for evaluating LoS activity/severity (A/S) and damage (D) by the physician (PhysGA-A, PhysGA-D) and the patient (PtGA-S, PtGA-D) over a one-month period. Scores range from 0 to 100. The GA scales were developed by paediatric LoS experts, on the basis of consensus agreement [3, 4, 8]. Whereas PhysGA-A and PtGA-S consider only cutaneous manifestations of the disease, PhysGA-D and PtGA-D take into account extracutaneous attributes/variables (physical disability, joint contracture, bone or skeletal muscle atrophy, eye involvement and central nervous system findings) as well.

Skindex-29 is a tool that measures the effects of skin disease on patients’ quality of life (QoL). It consists of 29 items arranged in three subscales: physical symptoms (7 items), emotions (10 items) and functioning (daily activities, fulfilling social roles and interpersonal interactions; 12 items), all scored from 0 to 100. This instrument is a valid and reliable tool and has been used as an outcome measure in clinical trials, for example in studies of therapies for atopic dermatitis, acne vulgaris and psoriasis [12–18]. A validated Polish version of the Skindex-29 questionnaire [13] was used with permission from Janowski Konrad, Ph.D.

### Psychometric assessment

Clinical signs of activity and damage in LoS patients were assessed with the use of LoSCAT and GA scales by two dermatology residents at the Medical University of Silesia independently. Before the initiation of the study, both examiners participated in an intensive training course carried out by an expert in LoS (ALŚ). Appendices describing each LoSCAT domain were prepared, with pictures of the example lesions added when possible. Moreover, the features determining disease activity and damage in adult patients with LoS were identified to properly evaluate the PhysGA-A and PhysGA-D. Detailed information about LoS and its natural course was prepared and this information was made available to patients previously received a diagnosis of LoS. Features determining disease severity and damage, as well as instructions for responding to the PtGA-S and PtGA-D, were described to them in Polish.

Analysis was restricted to the convergent validity and reliability of the mLoSSI, LoSDI and each of their domains. Because there is no alternative ‘gold standard’ instrument for the measurement of activity and damage in LoS, we evaluated the convergent validity of the LoSCAT by examining its relationship with GA. Convergent validity was assessed by comparing the mLoSSI (and its domains) to the Phys- and PtGA-A and by comparing the LoSDI (and its components) to the Phys and PtGA-D. Correlations of LoSCAT, PhysGA-A, PhysGA-D, PtGA-S and PtGA-D with three subscales (physical symptoms, emotions, functioning) of Skindex-29 were explored to allow for an additional analysis of the results.

To assess intra-rater reliability, all patients were examined twice by the same physician (ASS), who also completed the original English version of the LoSCAT and PhysGA-A and PhysGA-D, 48 h apart. All patients invited to participate in the validation survey were also asked to complete the PtGA-S and PtGA-D twice, with a 48-h interval. This period guaranteed that there would be no changes in the activity of LoS; at the same time, it was long enough to obtain memory annulment and to avert a carryover effect [19, 20].

To assess inter-rater reliability, all patients were evaluated and scored independently by two investigators (ASS, IRJ) with similar, a few years’ experience in the treatment of LoS.

### Statistical analysis

Because LoSCAT data are ordinal in nature, Cohen’s kappa statistic and interclass correlation coefficient were calculated to assess inter-rater reliability; Spearman’s correlation coefficient was used to assess intra-rater reliability. The strength of the agreement associated with kappa statistics was described as follows: poor (< 0), slight (0–0.2), fair (0.21–0.4), moderate (0.41–0.6), substantial (0.61–0.8) or almost perfect (0.81–1) [21]. Convergent validity of the LoSCAT and relationship of its components and VAS to QoL measures were determined using Spearman’s correlation coefficient. Spearman’s correlation coefficients were interpreted as follows: low positive (≥ 0.3, < 0.5), moderate (≥ 0.5, < 0.7), strong (≥ 0.7, < 0.9) or very strong (≥ 0.9) [22].

Statistica 12.0 (StatSoft, Inc., Tulsa, Oklahoma, USA) and PQStat (v.1.6.2; PQStat, Poznań, Poland) software was used for the statistical calculations.

## Results

The demographic and clinical characteristics of the 40 LoS patients included in the study are shown in Table 1.

LoS occurred predominantly in females in their fifth decade of life. The female:male ratio was 4.71:1. In only two patients (5%), the first symptoms appeared before the age of 18. Both of these patients were 34-year-old women (one had mixed LoS and the other had atrophoderma of Pasini and Pierini) who had disease onset in early childhood (5 and 4 years, respectively) and first relapse in adulthood (31 and 22 years, respectively). The most common LoS subtype was plaque. followed by Atrophoderma of Pasini and Pierini and the generalised subtypes. Mixed and linear subtypes were the rarest subtypes. Arthralgias affected 7.5% of patients overall (one with linear, one with mixed and one with generalised subtype of LoS). Of the comorbid medical conditions the most frequent were hypertensio arterialis, diabetes mellitus type 2 and gastroesophageal reflux. In fact, three patients had multiple disorders (patient 3: gastroesophageal reflux and psoriasis; patient 34: Still’s disease and vitiligo; patient 39: bronchial asthma and rheumatoid arthritis).

### Reliability

Table 2 summarises inter and intra-rater reliability of the LoSCAT and its components.

**Table 2:** Reliability of the Localized Scleroderma Cutaneous Assessment Tool components and global assessment of disease tools

	Inter-rater reliability		Intra-rater reliability
	Cohen’s kappa statistics^*^ (95% CI)	Intraclass correlation coefficient^*^ (95% CI)	Spearman’s correlation coefficient^*^ (95% CI)
mLoSSI	0.8936 (0.85, 0.93)	0,9937 (0.98, 0.99)	0,9739 (NA)
New lesion/lesion extension (N/E)	1 (1.00)	1 (1.00)	1 (NA)
Erythema (ER)	0.8096 (0.74, 0.88)	0.9862 (0.97, 0.99)	0.953 (NA)
Skin thickness (ST)	0.8991 (0.85, 0.95)	0.9932 (0.98, 0.99)	0.974 (NA)
LoSDI	0.8707 (0.82, 0.92)	0.9937 (0.98, 0.99)	0.9886 (NA)
Dermal atrophy (DAT)	0.9196 (0.87, 0.97)	0.9948 (0.99)	0.9741 (NA)
Subcutaneous atrophy (SAT)	0.9058 (0.86, 0.96)	0.9909 (0.98, 0.99)	0.9744 (NA)
Dyspigmentation (DP)	0.8227 (0.75, 0.90)	0.9746 (0.95, 0.98)	0.9823 (NA)
PhysGA-A	0.7406 (0.46, 092)	0.9647 (0.93, 0.98)	0.8939 (0.80, 0.94)
PhysGA-D	0.7109 (0.42, 0.91)	0.9532 (0.90, 0.98)	0.7118 (0.50, 0.84)

*CI* confidence interval, *mLoSSI* modified Localized Scleroderma Skin Severity Index, *LoSDI* Localized Scleroderma Damage Index, *PhysGA-A* physician global assessment of disease activity, *PhysGA-D* physician global assessment of disease damage

^*^p < 0.000001

#### Inter-rater reliability

The Cohen’s kappa statistics were 0.89 for the mLoSSI and 0.87 for the LoSDI, indicating almost perfect reliability. The Cohen’s kappa for components of the mLoSSI ranged from 0.81 for erythema and 0.90 for skin thickness to even 1 for new lesion/lesion extension domain, which reflects perfect agreement. Among components of the LoSDI, the lowest kappa was observed for dyspigmentation domain (0.82); for the others, it was higher (0.92 and 0.91 for dermal atrophy and subcutaneous atrophy, respectively).

For the PhysGA-A and PhysGA-D, the inter-rater reliability was substantial (0.74 and 0.71, respectively).

For the total scores of the mLoSSI and LoSDI, the intraclass correlation coefficients were almost perfect (> 0.99).

#### Intra-rater reliability

The Spearman’s rank correlation coefficient between two mLoSSI scores was 0.9739, indicating adequate reproducibility. An analysis of intra-rater reliability for each domain showed almost perfect reliability for all of them: new lesion/lesion extension erythema and skin thickness (r_s_ = 0.95–1). The intra-rater reliability of LoSDI and its domains was also almost perfect. The Spearman’s coefficient was high for LoSDI and its domains: dermal atrophy subcutaneous atrophy and dyspigmentation (r_s_ = 0.97–0.99). The intra-rater reliability of the PhysGA-D was substantial (0.71), and for the PhysGA-A it was almost perfect (0.89).

#### Convergent construct validity

Correlations between the LoSCAT and comparators are summarised in Table 3.

**Table 3:** Construct validity of the Localized Scleroderma Cutaneous Assessment Tool components with global assessment of disease tools

	PhysGA-A		PtGA-S	
	r_s_ (95% CI)	p	r_s_ (95% CI)	p
mLoSSI	0.64 (0.39, 0.79)	< 0.01	0.46 (0.16, 0.68)	< 0.01
New lesion / lesion extension (N/E)	0.68 (0.45, 0.81)	< 0.01	0.3 (−0.02, 0.56)	0.06
Erythema (ER)	0.53 (0.25, 0.72)	< 0.01	0.42 (0.11, 0.65)	< 0.01
Skin thickness (ST)	0.47 (0.18, 0.69)	< 0.01	0.39 (0.07, 0.62)	< 0.01
	PhysGA-D		PtGA-D	
	r_s_ (95% CI)	p	r_s_ (95% CI)	p
LoSDI	0.47 (0.17, 0.68)	< 0.01	0.24 (−0.09, 0.51)	0.14
Dermal atrophy (DAT)	0.33 (0.01, 0.58)	0.04	0.29 (−0.03, 0.55)	0.07
Subcutaneous atrophy (SAT)	0.44 (0.14, 0.66)	< 0.01	0.18 (−0.15, 0.47)	0.27
Dyspigmentation (DP)	0.42 (0.10, 0.64)	< 0.01	0.1 (−0.22, 0.40)	0.54

*CI* confidence interval, *mLoSSI* modified Localized Scleroderma Skin Severity Index, *PhysGA-A* physician global assessment of disease activity, *PtGA-S* patient global assessment of disease severity, *LoSDI* Localized Scleroderma Damage Index, *PhysGA-D* physician global assessment of disease damage, *PtGA-D* patient global assessment of disease damage, *r*_*s*_ Spearman’s rank correlation coefficient, *p p*-value

#### mLoSSI

The Spearman’s correlation coefficient indicated a moderate correlation between mLoSSI and PhysGA-A (r_s_ > 0.6, *p* < 0.01). All domains of mLoSSI had low positive correlations with PhysGA-A (r_s_ = 0.47–0.68, *p* < 0.01).. Similarly, low positive associations between mLoSSI and its domains – erythema score skin thickness score – as well as the PtGA-S were observed (r_s_ = 0.39–0.46, *p* < 0.01). The new lesion/lesion extension domain did not influence the PtGA-S score. A moderate correlation was found between PhysGA-A and PtGA-S (r_s_ = 0.65, *p* < 0.01).

#### LoSDI

A low positive correlation was observed between the PhysGA-D and LoSDI (r_s_ = 0.47, *p* < 0.01) and between the PhysGA-D and the LoSDI domains (r_s_ = 0.33–0.44, all statistically significant). A similar level of correlation was found between the PhysGA-D and PtGA-D (r_s_ = 0.42, *p* = 0.01). There was a tendency to positive correlation between the LoSDI, its domains, and the PtGA-D (r_s_ = 0.1–0.29, *p* = 0.07–0.54).

#### Correlations of LoSCAT and VAS with Skindex-29

Relationships between the physician- and patient-derivied measures and QoL are presented in Table 4. No correlation was found between mLoSSI and Skindex-29. The latter also did not correlate with PhysGA-A and PtGA-S. Increasing damage of the disease assessed by physician and patients on VAS correlated with worse QoL. A low positive correlation of all Skindex-29 subscales with PhysGA-D and PtGA-D was found. The LoSDI was not related to the results of the QoL assessment.

**Table 4:** Construct validity of clinical tools for assessing localized scleroderma with Skindex-29 subscales

	Skindex-29: emotions		Skindex-29: symptoms		Skindex-29: functioning	
	r_s_	p	r_s_	p	r_s_	p
mLoSSI	−0.05	0.76	−0.20	0.21	0.09	0.6
PhysGA-A	−0.13	0.44	0.04	0.81	0.07	0.65
PtGA-S	0.16	0.33	0.27	0.33	0.24	0.14
LoSDI	0.12	0.45	0.02	0.93	0.15	0.36
PhysGA-D	0.32	0.04	0.44	< 0.01	0.37	0.02
PtGA-D	0.4	< 0.01	0.48	< 0.01	0.38	< 0.01

*mLoSSI* modified Localized Scleroderma Skin Severity Index, *PhysGA-A* physician global assessment of disease activity, *PtGA-S* patient global assessment of disease severity, *LoSDI* Localized Scleroderma Damage Index, *PhysGA-D* physician global assessment of disease damage, *PtGA-D* patient global assessment of disease damage, *r*_*s*_ Spearman’s rank correlation coefficient, *p p*-value

## Discussion

Physician reported measures seem to provide the most adequate assessment of LoS and are the easiest way to monitor treatment in both everyday clinical practice and clinical trials. However, an ideal scoring system was not available until relatively recently. In 2008–2014 Arkachaisri et al. developed and initially validated in paediatric patients the LoSCAT, a clinical skin score for the assessment of disease severity and damage in LoS [3, 4, 8]. It is considered a reliable and valid tool for assessing LoS in children [2–4].

We conducted a cross-sectional study with adult LoS patients to determine the convergent validity in addition to inter- and intra-examiner reliability of the LoSCAT. The LoSCAT was completed independently by two physicians and one of them completed it again after 48 h. Because LoS is a slow, progressive disorder, disease status is unlikely to change within this short period; however, the period seems to be is long enough to avoid recall bias [19, 20]. There was excellent agreement among repeated scores of the LoSCAT performed by a single rater (intra-rater reliability) as well as among raters (inter-rater reliability). The almost perfect intra- and inter-rater agreement was unsurprising, and we think it resulted from intensive training and preparation of an appendices to the LoSCAT before the study started. Charts describing the mLoSSI and LoSDI domains accompanied by photographs of real patients’ lesions offered rather good clues during each patient’s assessment. This kind of ‘LoSCAT instruction’ can be used even by inexperienced examiners, improving the results of disease evaluation. Thus, we reduced the effect of experience on inter-rater reliability. The PhysGA-D had only substantial inter- and intra-rater reliability, indicating that it has is less reliable than the LoSDI in the assessment of damage in adults with LoS.

When comparing correlation coefficients between the LoSCAT and other scales from the present study with validation studies in children with LoS, relatively similar patterns were identified. We observed a moderate correlation between mLoSSI and GA of disease activity/severity, confirming satisfactory convergence of analysed measures, as was the case in a study by Arkachaisri et al. [3]. The lack of association between PtGA-S and new lesions appearing or the enlargement of an existing lesion may seem surprising. A likely explanation is the patient’s understanding of the chronic nature of the disease and the recurrent character of the lesions (in almost all cases).

A low positive correlation was found between two clinician-reported measures, the LoSDI and PhysGA-D. As in a previous study conducted in a group of children with LoS, the physician reported LoSDI was not associated with patient derived PtGA-D scores [4]. This finding may be due to the fact that the LoSDI includes neither extracutaneous manifestation nor psychosocial condition assessment, in contrast to the GA-D [4]. Additionally the relationships of mLoSSI and LoSDI with Skindex-29 were assessed in our study and no association was found between higher activity or damage of the LoS and QoL. Similarly, the correlation of QoL with mLoSSI and LoSDI was poor and nonsignificant, when Children Dermatology Life Quality Index was assessed by Arkachaisri et al. [3, 4]. Cutaneous activity (LoSSI) and damage (LoSDI) did not influence the QoL in children also in other studies [23, 26, 27]. Although Klimas et al. found correlation between Skindex-29 and mLoSSI, specifically the symptoms domain, LoSDI did not correlate with Skindex-29 in their Morphea in Adults and Children cohort [24]. Satisfactory correlation of the PhysGA-D and PtGA-D with all Skindex-29 components in our study may indicate that Skindex-29 is a proper dermatology-specific tool for assessment of a QoL in adult LoS patients. The need to use additional health status instruments due to shortcomings of the LoSCAT was signalled in our earlier paper [25]. Adult patients with LoS scored lower on QoL than those with paediatric-onset LoS [25, 26]. Moreover, the recently published study proposed that physicians evaluate impairment of activities of daily living (ADL), not only QOL, in LoS patients [27]. Multiple affected body parts and leg lesions significantly impaired ADL of LoS patients, such as feeding, mobility (on level surface) and stairs [27]. A holistic approach to LoS patients is extremely important. Therefore, measurement tools related to the physical, mental, emotional and social functioning of patients seem to be important for an assessment of adult-onset LoS, but as far as psychometric properties, the QOL measures and LoS disability score were should not be used as a convergent criteria on validity of the LoSCAT. While GAs are partially based on variables that are within LoSCAT measures (i.e. erythema, skin thickness, dyspigmentation), QoL or ADL patient reports are different concepts.

The results demonstrate excellent reliability and convergent construct validity of the LoSCAT, providing evidence of its value in clinical trials with adult-onset LoS. In our opinion LoSCAT seems to be relevant, applicable, and easy to understand and use. It is sufficiently discriminating and comprehensive to cover the various clinical manifestations of LoS. At the same time, it is neither complicated nor time-consuming, which are crucial issues in a scoring system for routine daily use. In light of the results, it seems the LoSCAT may become the most popular instrument for the assessment of LoS skin activity and damage. Further prospective research should investigate other psychometric properties of the scale such as sensitivity to change in adult population.

## Conclusions

Even though the LoSCAT had satisfactory convergence validity and excellent reliability in the assessment of cutaneous lesions, additional health status instruments should be included in a future core set of outcome measures for LoS clinical trials in adult patients.

## Abbreviations

ADL: Activities of daily living
GA: Global Assessment
LoS: Localized scleroderma
LoSCAT: Localized Scleroderma Cutaneous Assessment Tool
LoSDI: Localized Scleroderma Skin Damage Index
mLoSSI: Modified Localized Scleroderma Skin Severity Index
PhysGA-A: Physician Global Assessment of Disease Activity
PhysGA-D: Physician Global Assessment of Disease Damage
PtGA-D: Patient Global Assessment of Disease Damage
PtGA-S: Patient Global Assessment of Disease Severity
QoL: Quality of life
r_s_: Spearman’s Correlation Coefficient
VAS: Visual Analogue Scale
